# Supplementary material for: BET inhibition disrupts transcription but retains enhancer-promoter contact
Source: Nat Commun. 2021 Jan 11;12:223. doi: 10.1038/s41467-020-20400-z (PMC7801379; doi:10.1038/s41467-020-20400-z)
Supplement: Supplementary file 1 — Supplementary Information [file 41467_2020_20400_MOESM1_ESM.pdf]

BET inhibition disrupts transcription but retains enhancer-promoter contact

Crump et al.

Supplementary Information

Supplementary Figure 1

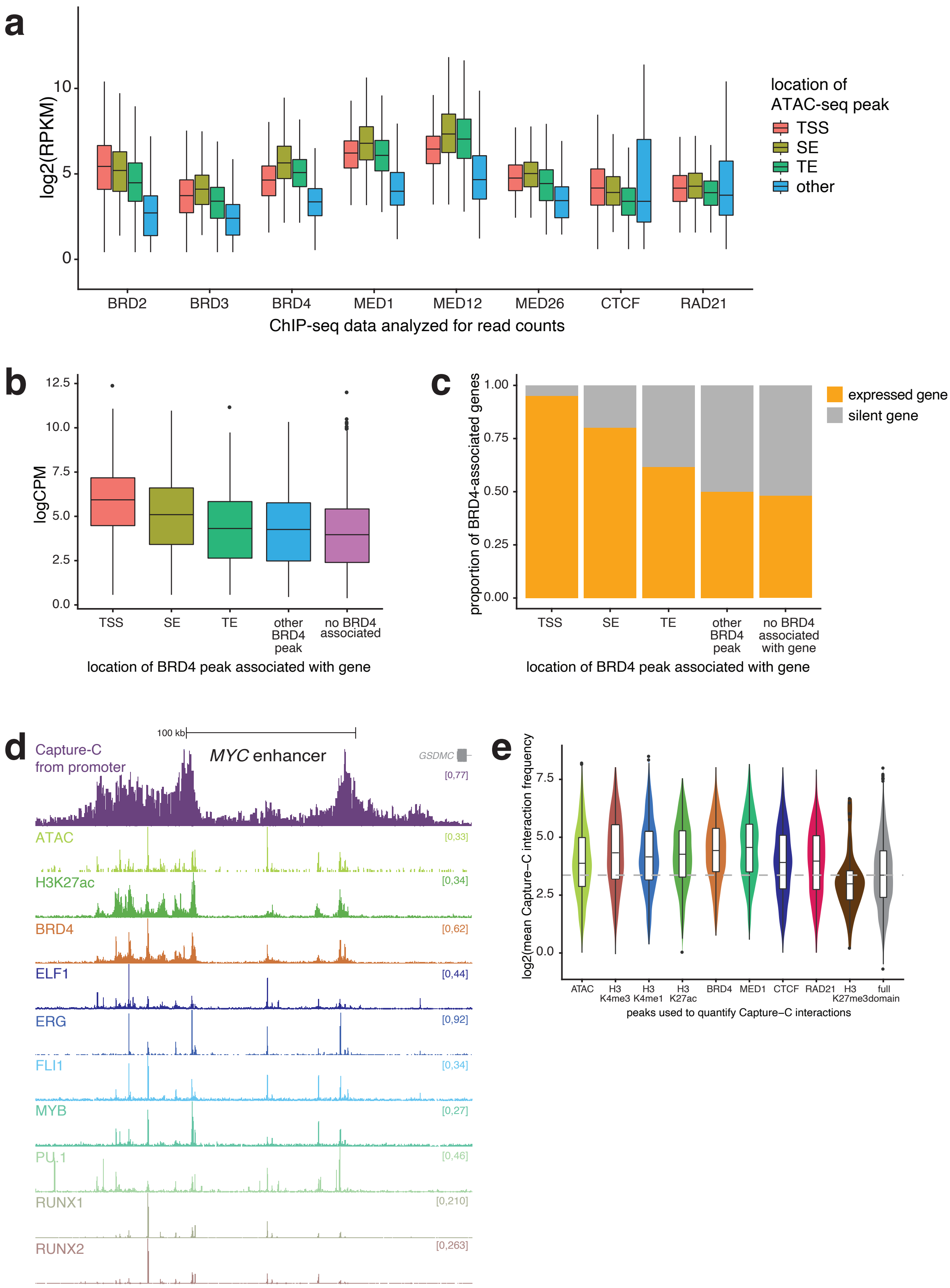

Supplementary Figure 1.

a Boxplot showing RPKM (reads per kb per million reads) levels of BET proteins, Mediator subunits, CTCF and RAD21 at ATAC peaks found at TSSs (red), within super-enhancers (SE; olive), within typical enhancers (TE: green) or at other sites (blue) in SEM cells. Midline shows median, with upper and lower hinges showing 25th and 75th percentile, respectively. Upper and lower hinges extend to the largest and smallest datapoints within 1.5 times the interquartile range of either hinge. Analysis of data from one experiment. **b** Level of expression of genes (logCPM (counts per million) from nascent RNA-seq), classified based on the location of the nearest BRD4 peak. Midline shows median, with upper and lower hinges showing 25th and 75th percentile, respectively. Upper and lower hinges extend to the largest and smallest datapoints within 1.5 times the interquartile range of either hinge; outliers are plotted as dots. Analysis of data from one experiment. **c** Proportion of expressed genes, classified based on the location of the nearest BRD4 peak to each gene. **d** Transcription factor ChIP-seq tracks at the *MYC* enhancer. Capture-C from the *MYC* promoter (mean of three replicates), ATAC-seq and H3K27ac and BRD4 ChIP-seq tracks are reproduced from Fig 1c for comparison. **e** Frequency of Capture-C promoter interactions at 10 kb regions flanking ATAC-seq/ChIP-seq peaks for the indicated antibodies. Violin plot shows frequency distribution. Midline shows median, with upper and lower hinges showing 25th and 75th percentile, respectively. Upper and lower hinges extend to the largest and smallest datapoints within 1.5 times the interquartile range of either hinge; outliers are plotted as dots. Analysis of data from three independent experiments. Full domain: data for the entire analyzed regions divided into 10 kb bins.

Supplementary Figure 2

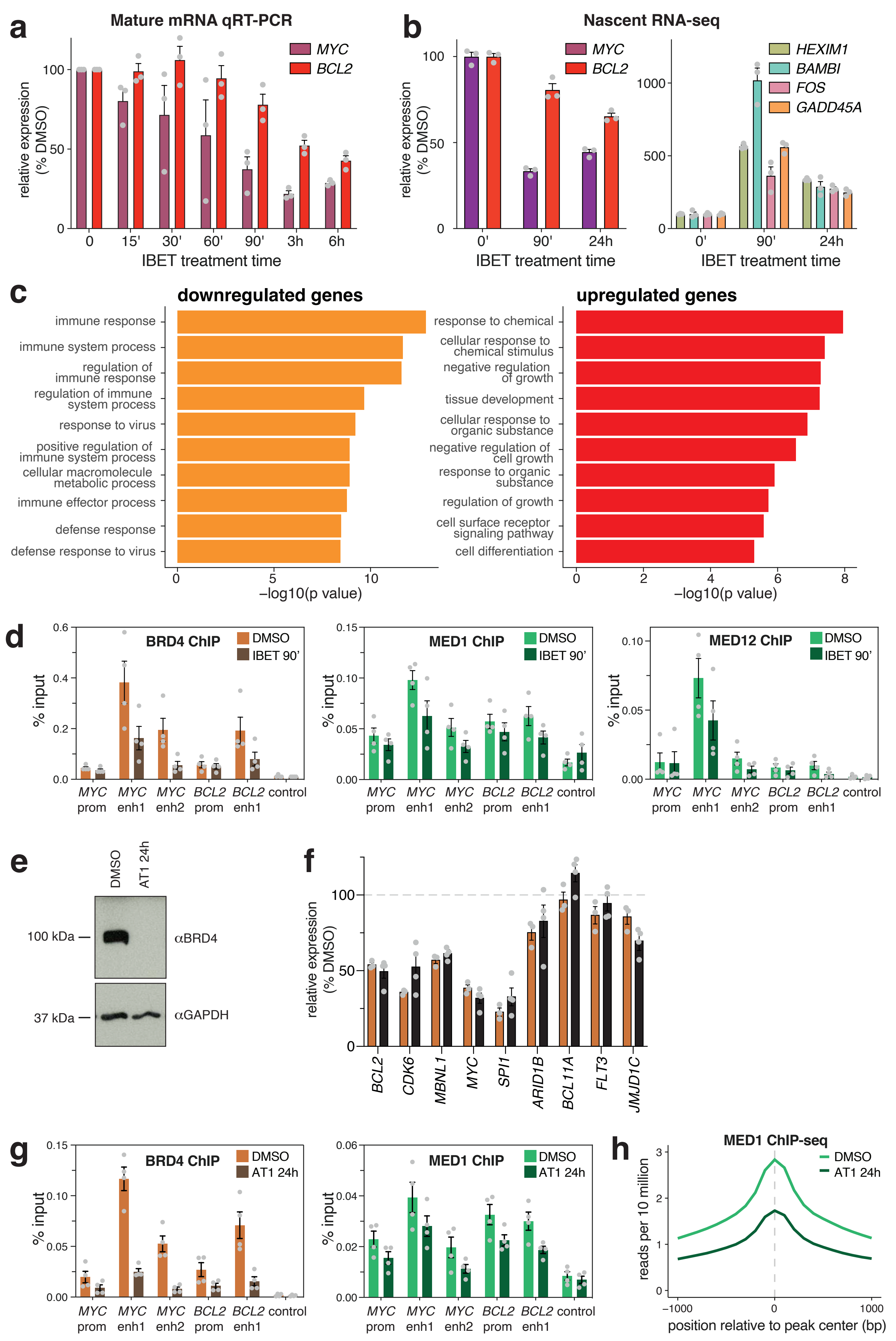

Supplementary Figure 2.

a qRT-PCR analysis of RNA levels following 1 μM IBET-151 treatment for the indicated times, using mature mRNA PCR primers. Values are normalized to *YWHAZ* mature mRNA levels, relative to DMSO treatment. Mean of three biological replicates, normalized to expression in DMSO; error bars show SEM. Source data are provided as a Source Data file. **b** Quantification of nascent RNA-seq levels of genes shown in Fig 2a, following 90 min or 24h IBET treatment. Data are CPM-normalized, relative to expression levels under DMSO treatment. Mean of three biological replicates; error bars show SEM. Source data are provided as a Source Data file. **c** Top ten biological pathway GO terms most significantly enriched in down- and upregulated genes (logFC <-1 or >1, FDR <0.05) following 90 min IBET treatment. **d** ChIP-qPCR for BRD4, MED1 and MED12 following 90 min treatment with DMSO (light) or IBET (dark). Mean of four biological replicates; error bars show SEM. Primer locations are shown in Fig 1c, d. Source data are provided as a Source Data file. **e** Western blot of BRD4 and GAPDH following treatment with DMSO or 1 μM AT1 for 24h. Representative of three biological replicates. Uncropped blots are available as a Source Data file. **f** qRT-PCR analysis of RNA levels following 1 μM IBET (orange) or AT1 (brown) treatment for 24h, using mature mRNA PCR primers. Values are normalized to *YWHAZ* mature mRNA levels, relative to 24h DMSO treatment. n=3 biological replicates for IBET data, n=4 for AT1 data; mean normalized to expression in DMSO; error bars show SEM. Source data are provided as a Source Data file. **g** ChIP-qPCR for BRD4 and MED1 following 24h treatment with DMSO (light) or AT1 (dark). Mean of four biological replicates; error bars show SEM. Primer locations are shown in Fig 1c-d. Source data are provided as a Source Data file. **h** Metaplot of reference-normalized mean MED1 levels at BRD4 peaks in SEM cells treated with DMSO (light green) or AT1 (dark green) for 24h.

Supplementary Figure 3

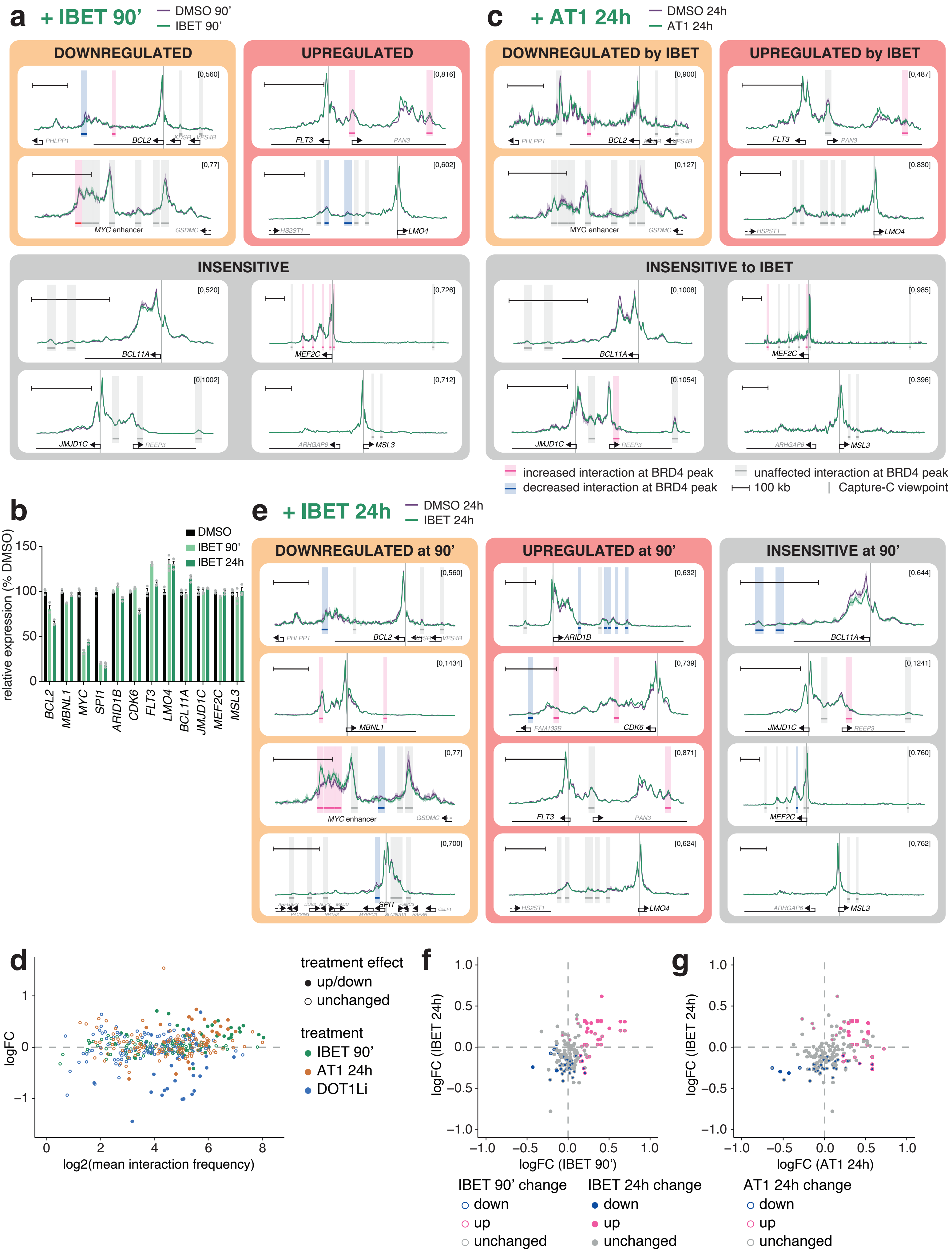

Supplementary Figure 3.

a Capture-C traces at genes which are transcriptionally downregulated, upregulated or unaffected following 90 min IBET treatment. Purple line shows the profile in DMSO-treated cells; green line is from cells treated with IBET for 90 min; ribbon shows +/-1 SD for three replicates. Vertical gray bar indicates the capture point for each gene. Horizontal bars show 10 kb region around BRD4 ChIP-seq peaks. Shading highlights effect of IBET 90 min treatment on promoter interaction frequency within that window: pink bars indicate statistically-significant increases; blue bars indicate decreases; gray bars indicate no significant difference (Holm-Bonferroni adjusted p-value <0.05, paired Mann-Whitney test; adjusted P values are given in Supplementary Data 3). Scale bar shows 100 kb. **b** Quantification of nascent RNA-seq expression of genes shown in (a) and Fig 3e, following 90 min or 24h IBET treatment. Data are CPM-normalized, relative to expression levels under DMSO treatment, mean of three biological replicates; error bars show SEM. Source data are provided as a Source Data file. **c** Capture-C traces at genes which are transcriptionally downregulated, upregulated or unaffected following 90 min IBET treatment. Purple line shows the profile in DMSO-treated cells; green line is from cells treated with AT1 for 24h; data are displayed as in (a). **d** MA plot of changes in Capture-C BRD4 peak interaction with promoters following IBET 90 min (green), AT1 24h (orange) or DOT1Li (blue) treatment. Significantly changed interactions (Holm-Bonferroni adjusted p-value <0.05, paired Mann-Whitney test; adjusted P values are given in Supplementary Data 3) are shown as closed circles; non-significant changes are open circles. **e** Capture-C traces at genes which are transcriptionally downregulated, upregulated or unaffected following 90 min IBET treatment. Purple line shows the profile in DMSO-treated cells; green line is from cells treated with IBET for 24h; data are displayed as in (a). **f** Comparison of changes in Capture-C promoter interactions following IBET treatment for 90 min or 24h. Mean of three biological replicates. Outer color indicates the effect of 90 min IBET treatment on interaction at each BRD4 peak, inner color indicates the effect of 24h treatment. Blue: decreased interaction; pink: increased interaction; gray: no change in interaction (Holm-Bonferroni adjusted p-value <0.05, paired Mann-Whitney test; adjusted P values are given in Supplementary Data 3). **g** Comparison of changes in Capture-C promoter

interactions following IBET (outer color) or AT1 treatment (inner color) for 24h. Data are displayed as in (f).

Supplementary Figure 4

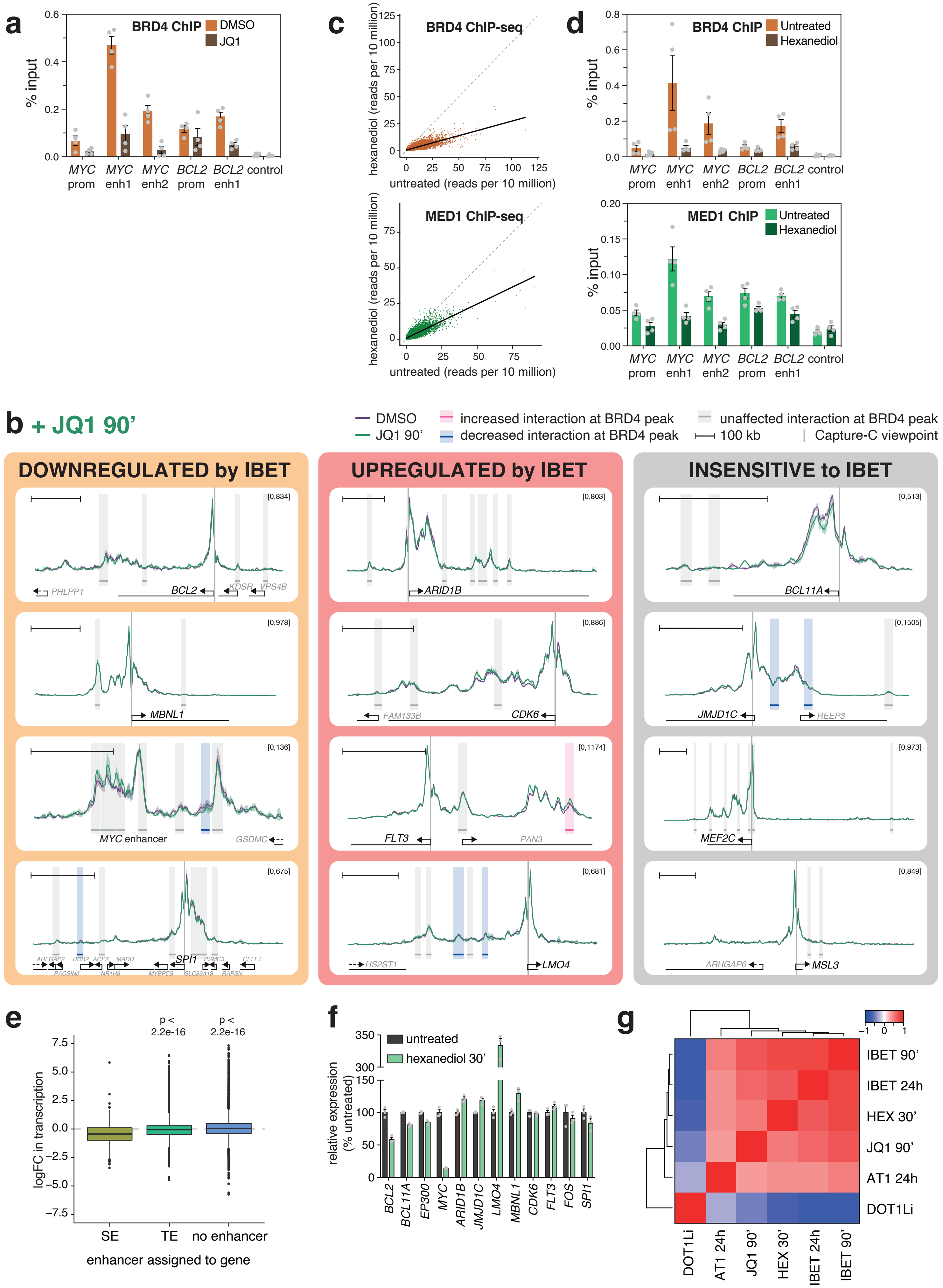

Supplementary Figure 4.

a ChIP-qPCR for BRD4 following 90 min treatment with DMSO (orange) or 1 µM JQ1 (brown). Mean of five biological replicates; error bars show SEM. Primer locations are shown in Fig 1c-d. Source data are provided as a Source Data file. **b** Capture-C traces at genes which are transcriptionally downregulated, upregulated or unaffected following 90 min IBET treatment. Purple line shows the profile in DMSO-treated cells; green line is from cells treated with JQ1 for 90 min; data are displayed as in Supplementary Fig 3a. **c** Reference-normalized BRD4 (orange) and MED1 (green) ChIP-seq reads at BRD4 peaks from untreated SEM cells (x-axis) or cells treated with 1,6-hexanediol for 30 min (y-axis). Solid line shows data trend (generalized additive model). Source data are provided as a Source Data file. **d** ChIP-qPCR for BRD4 and MED1 from untreated SEM cells (light) or cells treated with 1,6-hexanediol for 30 min (dark). Mean of four biological replicates; error bars show SEM. Primer locations are shown in Fig 1c-d. **e** Effect of hexanediol treatment on gene expression, classified based on the type of enhancer associated with each gene. SE: super-enhancer; TE: typical enhancer. p values indicate the statistical significance of the difference in logFC of each gene set compared to SE-associated genes (Wilcoxon rank sum test; TE-associated genes $p<2.2x10^{-16}$; genes not annotated with an enhancer $p<2.2x10^{-16}$). Boxplot midline shows median, with upper and lower hinges showing 25th and 75th percentile, respectively. Upper and lower hinges extend to the largest and smallest datapoints within 1.5 times the interquartile range of either hinge; outliers are plotted as dots. Mean of three independent experiments. **f** Quantification of nascent RNA-seq expression of genes shown in Fig 4g, following 30 min 1,6-hexanediol treatment. Data are CPM-normalized, relative to expression levels in untreated cells, mean of three biological replicates; error bars show SEM. **g** Pearson correlation of the changes in interaction frequency (logFC) between BRD4 peaks and promoters (10 kb windows) following the indicated treatments. Dendrogram shows hierarchical clustering of datasets. Source data are provided as a Source Data file.

Supplementary Figure 5

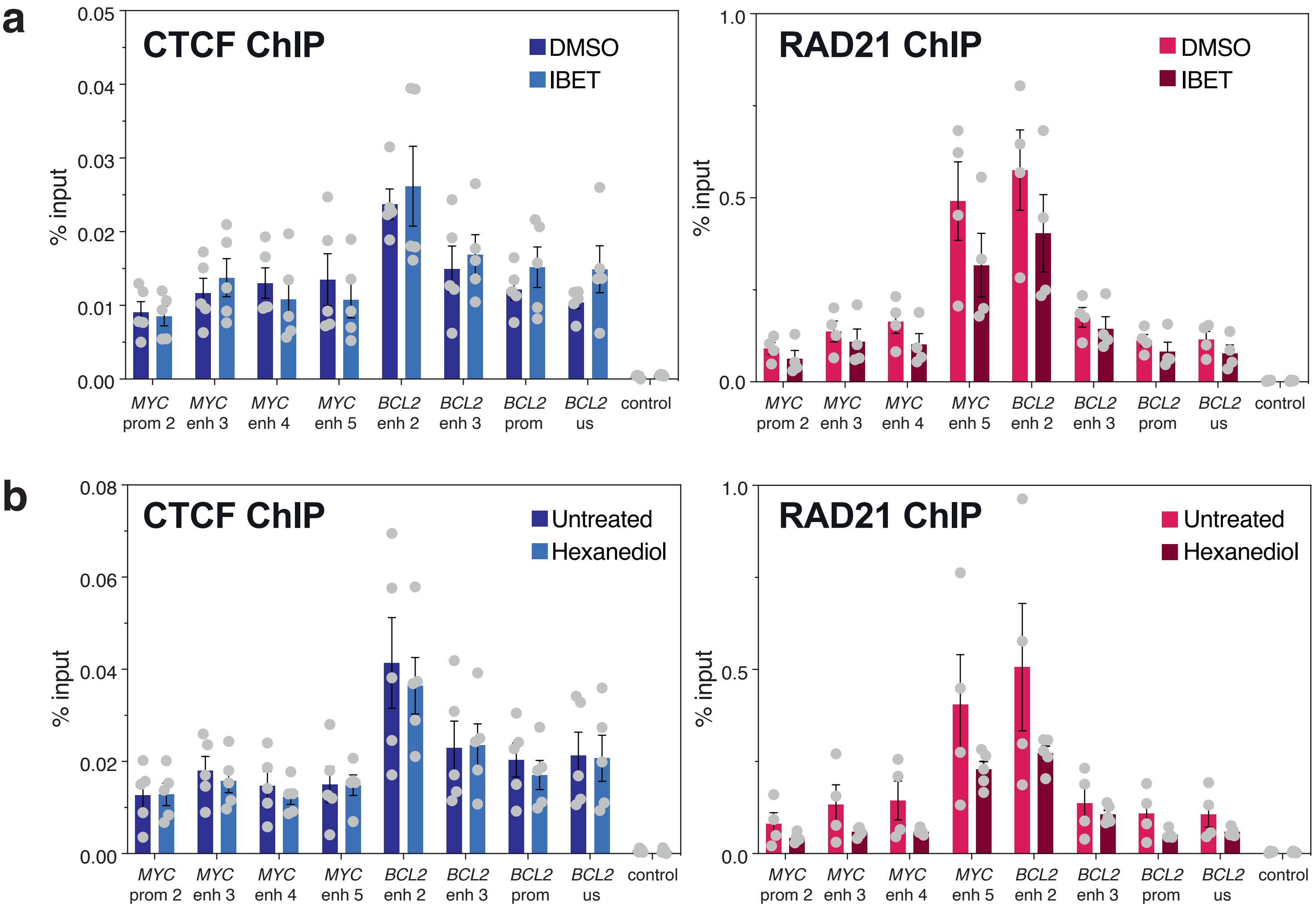

Supplementary Figure 5.

a ChIP-qPCR for CTCF and RAD21 in SEM cells following 90 min treatment with DMSO or 1 μM IBET. Mean of four (RAD21) or five (CTCF) biological replicates; error bars show SEM. Source data are provided as a Source Data file. **b** ChIP-qPCR for CTCF and RAD21 from untreated SEM cells or following 30 min treatment with 1.5 % 1,6-hexanediol. Mean of four (RAD21) or five (CTCF) biological replicates; error bars show SEM. Source data are provided as a Source Data file.

Supplementary Table 1 Antibodies used in this study

**Target**	**Catalogue Number and Batch**	**Company**	**Amount used per 10^7 cells for ChIP/ WB dilution**
BRD2	A302-582A lot 1	Bethyl	2 μg
BRD3	A302-368A lot 1	Bethyl	1 μg
BRD4 (ChIP)	A301-985A lot 1	Bethyl	2 μg
BRD4 (Western)	ab128874 lot GR3251918-5	Abcam	1:10000
MED1	A300-793A lot 8	Bethyl	2 μg
MED12	A300-774A lot 1	Bethyl	2 μg
MED26	A302-370A lot 1	Bethyl	2 μg
RAD21	ab992 lot GR3253930-3	Abcam	2 μg
CTCF	07-729 lot 2922425	Millipore	2 μl
GAPDH	A300-641A lot 3	Bethyl	1:20000

Supplementary Table 2 PCR Primers used in this study

Primer name	Forward Sequence/ TaqMan probe code	Reverse Sequence
MYC (mature)	Hs0015348_m1	
MYC (intronic)	AAGGGAGGCGAGGATGTGTCC	GGCTGGGTGCGGAGATTCG
BCL2 (mature)	Hs00608023_m1	
BCL2 (intronic)	CGATAACGCCTGCCATCTAA	CCACCACATCCTACTGGATTAC
HEXIM1	Hs00538918_s1	
BAMBI	Hs03044164_m1	
FOS	Hs00170630_m1	
GADD45A	Hs00169255_m1	
YWHAZ	Hs03044281_g1	
MYC prom	TGCGGGCGTCCTGGGAAG	GTGGATGCGGCAAGGGTTG
MYC enh 1	CTTGAGAGGCCAAGCATCA	CTCCTTTGTGTGCAGAGTTCTA
MYC enh 2	AGCAGACACACAATGGATAGG	CCTCTGAAAGGAGAAGTGAGTTAG
BCL2 prom	GTTCAGGTACTCAGTCATCCAC	GGAGGATTGTGGCCTTCTTT
BCL2 enh 1	GAGCCCTCAACCTTGTGATAG	AAGGTAGCCCTGACCATAGA
Negative control	GGCTCCTGTAACCAACCACTACC	CCTCTGGGCTGGCTTCATTC
MYC prom 2	TCCTCCAGTAACTCCTCTTTCT	GGACAGGCGGTTCCTTAAA
MYC enh 3	GATATGGACTTCAGCAGTGACTC	GTTCCTACAGTGGCTGGTTTAT
MYC enh 4	GCTCAAATTGCCCTAACTTCAC	GGCGACTATGGGATAGCATTTA
MYC enh 5	TCTTTCCAGAGCAGCATTCC	AGGCAGTAGATGGCAGTAGA
BCL2 enh 2	GAGAAGAAACGACGCGAAGG	GGTTTCAGCGGCTCCAAATA
BCL2 enh 3	TGTTGCCTTTAGGCTGTTCT	TCTCACCTGCTCGCTCTAA

Supplementary Table 3 Publicly available datasets used in this study

Data type	Cell type	Sample	GEO accession number
ATAC-seq	SEM	Control	GSE117865 [https://www.ncbi.nlm.nih.gov/geo/query/acc.cgi?acc=GSE117865]
Capture-C	SEM	DMSO 7d	GSE117865 [https://www.ncbi.nlm.nih.gov/geo/query/acc.cgi?acc=GSE117865]
Capture-C	SEM	EPZ-5676 7d	GSE117865 [https://www.ncbi.nlm.nih.gov/geo/query/acc.cgi?acc=GSE117865]
Capture-C	SEM CTCF-miniAID Clone 27	Untreated	GSE121257 [https://www.ncbi.nlm.nih.gov/geo/query/acc.cgi?acc=GSE121257]
Capture-C	SEM CTCF-miniAID Clone 27	IAA 48h	GSE121257 [https://www.ncbi.nlm.nih.gov/geo/query/acc.cgi?acc=GSE121257]
Capture-C	SEM CTCF-miniAID Clone 35	Untreated	GSE121257 [https://www.ncbi.nlm.nih.gov/geo/query/acc.cgi?acc=GSE121257]
Capture-C	SEM CTCF-miniAID Clone 35	IAA 48h	GSE121257 [https://www.ncbi.nlm.nih.gov/geo/query/acc.cgi?acc=GSE121257]
ChIP-seq	SEM	H3K4me1	GSE74812 [https://www.ncbi.nlm.nih.gov/geo/query/acc.cgi?acc=GSE74812]
ChIP-seq	SEM	H3K4me3	GSE74812 [https://www.ncbi.nlm.nih.gov/geo/query/acc.cgi?acc=GSE74812]
ChIP-seq	SEM	H3K27ac	GSE74812 [https://www.ncbi.nlm.nih.gov/geo/query/acc.cgi?acc=GSE74812]
ChIP-seq	SEM	BRD4	GSE83671 [https://www.ncbi.nlm.nih.gov/geo/query/acc.cgi?acc=GSE83671]
ChIP-seq	SEM	MED1	GSE83671 [https://www.ncbi.nlm.nih.gov/geo/query/acc.cgi?acc=GSE83671]
ChIP-seq	SEM	CTCF	GSE117865 [https://www.ncbi.nlm.nih.gov/geo/query/acc.cgi?acc=GSE117865]
ChIP-seq	SEM	ELF1	GSE117865 [https://www.ncbi.nlm.nih.gov/geo/query/acc.cgi?acc=GSE117865]

ChIP-seq	SEM	ERG	GSE117865 [https://www.ncbi.nlm.nih.gov/geo/query/acc.cgi?acc=GSE117865]
ChIP-seq	SEM	FLI1	GSE117865 [https://www.ncbi.nlm.nih.gov/geo/query/acc.cgi?acc=GSE117865]
ChIP-seq	SEM	MYB	GSE117865 [https://www.ncbi.nlm.nih.gov/geo/query/acc.cgi?acc=GSE117865]
ChIP-seq	SEM	RUNX1	GSE42075 [https://www.ncbi.nlm.nih.gov/geo/query/acc.cgi?acc=GSE42075]
ChIP-seq	SEM	RUNX2	GSE117865 [https://www.ncbi.nlm.nih.gov/geo/query/acc.cgi?acc=GSE117865]
ChIP-seq	SEM	SPI1	GSE117865 [https://www.ncbi.nlm.nih.gov/geo/query/acc.cgi?acc=GSE117865]
