# Supplementary material for: BET inhibition disrupts transcription but retains enhancer-promoter contact
Source: Nat Commun. 2021 Jan 11;12:223. doi: 10.1038/s41467-020-20400-z (PMC7801379; doi:10.1038/s41467-020-20400-z)
Supplement: Supplementary file 3 — Description of Additional Supplementary Files [file 41467_2020_20400_MOESM3_ESM.pdf]

Description of Additional Supplementary Files

File Name: Supplementary Data 1
Description: Capture-C probes used in this study

File Name: Supplementary Data 2
Description: Nascent RNA-seq data following treatment with 1 μM IBET-151 for 90 min or 24h or 1.5 % 1,6-hexanediol for 30 min

File Name: Supplementary Data 3
Description: Capture-C statistics for interactions between capture probe DpnII fragments and BRD4 peaks, following treatment with 1 μM IBET-151 for 90 min or 24h, 1 μM AT1 for 24h, 1 μM JQ1 for 90 min, 1.5 % 1,6-hexanediol for 30 min or 2 μM EPZ-5676 (DOT1Li) for 7d.
