# Supplementary material for: BET inhibition disrupts transcription but retains enhancer-promoter contact
Source: Nat Commun. 2021 Jan 11;12:223. doi: 10.1038/s41467-020-20400-z (PMC7801379; doi:10.1038/s41467-020-20400-z)
Supplement: Supplementary file 7 — Reporting Summary [file 41467_2020_20400_MOESM7_ESM.pdf]

nature research

Corresponding author(s): Thomas A Milne

Last updated by author(s): Nov 10, 2020

Reporting Summary

Nature Research wishes to improve the reproducibility of the work that we publish. This form provides structure for consistency and transparency in reporting. For further information on Nature Research policies, see Authors & Referees and the Editorial Policy Checklist .

Statistics

For all statistical analyses, confirm that the following items are present in the figure legend, table legend, main text, or Methods section.

n/a	Confirmed	
☐	☒	The exact sample size (*n*) for each experimental group/condition, given as a discrete number and unit of measurement
☐	☒	A statement on whether measurements were taken from distinct samples or whether the same sample was measured repeatedly
☐	☒	The statistical test(s) used AND whether they are one- or two-sided<br>*Only common tests should be described solely by name; describe more complex techniques in the Methods section.*
☐	☒	A description of all covariates tested
☐	☒	A description of any assumptions or corrections, such as tests of normality and adjustment for multiple comparisons
☐	☒	A full description of the statistical parameters including central tendency (e.g. means) or other basic estimates (e.g. regression coefficient) AND variation (e.g. standard deviation) or associated estimates of uncertainty (e.g. confidence intervals)
☐	☒	For null hypothesis testing, the test statistic (e.g. *F*, *t*, *r*) with confidence intervals, effect sizes, degrees of freedom and *P* value noted<br>*Give P values as exact values whenever suitable.*
☒	☐	For Bayesian analysis, information on the choice of priors and Markov chain Monte Carlo settings
☒	☐	For hierarchical and complex designs, identification of the appropriate level for tests and full reporting of outcomes
☐	☒	Estimates of effect sizes (e.g. Cohen's *d*, Pearson's *r*), indicating how they were calculated

Our web collection on statistics for biologists contains articles on many of the points above.

Software and code

Policy information about availability of computer code

Data collection	Next-generation sequencing (Illumina). No software was used for data collection.
Data analysis	For ChIP-seq and ATAC-seq, quality control of FASTQ reads, genome alignment, PCR duplicate filtering, blacklisted region filtering and UCSC data hub generation was performed using the NGSeqBasic pipeline (https://github.com/Hughes-Genome-Group/NGseqBasic/releases). Directories of sequence tags (reads) were generated from the sam files using the Homer (v4.8) tool makeTagDirectory. The makeBigWig.pl command was used to generate bigwig files for visualisation in UCSC, normalising tag counts to tags per 10 million. Peaks were called using the Homer tool findPeaks, with the input track provided for background correction, using the -style histone or -style factor options to call peaks in histone modification or transcription factor/ATAC datasets, respectively.<br>For RNA-seq analysis, following QC analysis with fastQC v0.10.1 (http://www.bioinformatics.babraham.ac.uk/projects/fastqc) reads were aligned against the human genome assembly (hg19) using STAR (v2.4.2a). Duplicate reads were removed using the picard (v1.105) command MarkDuplicates.jar (http://broadinstitute.github.io/picard). Gene expression levels were quantified as read counts using the featureCounts function from the Subread package (v2.0.0) with default parameters. The read counts were used to identify differential gene expression between conditions and generate RPKM values using the edgeR package (v3.26.5).<br>Capture-C analysis was performed using scripts available at https://github.com/Hughes-Genome-Group/CCseqBasicF/releases

For manuscripts utilizing custom algorithms or software that are central to the research but not yet described in published literature, software must be made available to editors/reviewers. We strongly encourage code deposition in a community repository (e.g. GitHub). See the Nature Research guidelines for submitting code & software for further information.

Data

Policy information about availability of data

All manuscripts must include a data availability statement. This statement should provide the following information, where applicable:

- Accession codes, unique identifiers, or web links for publicly available datasets
- A list of figures that have associated raw data
- A description of any restrictions on data availability

All newly-generated high throughput data have been deposited in the Gene Expression Omnibus (GEO) under the accession number GSE139437 (https://www.ncbi.nlm.nih.gov/geo/query/acc.cgi?acc=gse139437).
Listed below are the datasets associated with each figure:
Figure 1: SEM ChIP-seq (BRD2, BRD3, MED1, MED12, MED26, RAD21), SEM Capture-C (DMSO 90 min in triplicate)
Figure 2: SEM Nascent RNA-seq (DMSO and IBET 90 min, DMSO and IBET 24h, all in triplicate), SEM ChIP-seq (BRD4 DMSO and IBET90 min, DMSO and AT1 24h, MED1 DMSO and AT1 24h)
Figure 3: SEM Capture-C (DMSO and IBET 90 min, DMSO and IBET 24h, DMSO and AT1 24h, all in triplicate), SEM Nascent RNA-seq (DMSO and IBET 90 min, both in triplicate)
Figure 4: SEM ChIP-seq (BRD4, MED1 untreated and 1,6-hexanediol 30 min), SEM Nascent RNA-seq (Untreated and 1,6-hexanediol 30 min, both in triplicate), SEM Capture-C (Untreated and 1,6-hexanediol 30 min, both in triplicate)
Figure 5: SEM Capture-C (DMSO 90 min, DMSO and AT1 24h, all in triplicate), SEM ChIP-seq (RAD21)
Supplementary Figure 1: SEM ChIP-seq (BRD2, BRD3, MED1, MED12, MED26, RAD21), SEM Capture-C (DMSO 90 min in triplicate)
Supplementary Figure 2: SEM Nascent RNA-seq (DMSO and IBET 90 min, DMSO and IBET 24h, all in triplicate), SEM ChIP-seq (MED1 DMSO and AT1 24h)
Supplementary Figure 3: SEM Capture-C (DMSO and IBET 90 min, DMSO and AT1 24h, DMSO and IBET 24h, all in triplicate), SEM Nascent RNA-seq (DMSO and IBET 90 min, DMSO and IBET 24h, all in triplicate)
Supplementary Figure 4: SEM Capture-C (DMSO and IBET 90 min, DMSO and IBET 24h, Untreated and 1,6-hexanediol 30 min, DMSO and AT1 24h, DMSO and JQ1 90 min, all in triplicate), SEM Nascent RNA-seq (Untreated and 1,6-hexanediol 30 min, both in triplicate)

Field-specific reporting

Please select the one below that is the best fit for your research. If you are not sure, read the appropriate sections before making your selection.

☒ Life sciences ☐ Behavioural & social sciences ☐ Ecological, evolutionary & environmental sciences

For a reference copy of the document with all sections, see nature.com/documents/nr-reporting-summary-flat.pdf

Life sciences study design

All studies must disclose on these points even when the disclosure is negative.

Sample size	Statistical methods were not used to assign sample size. Experiments were performed with 3-4 biological replicates as is common in the field; the observed biological effects of interest were consistent between replicates.
Data exclusions	No data were excluded from this analysis
Replication	ChIP-seq data represent a single biological replicate, with peaks and troughs of signal at specific loci confirmed by ChIP-qPCR. ChIP-qPCR experiments were conducted with multiple biological replicates to confirm any changes following drug treatment.<br>Capture-C experiments were conducted in triplicate, with averaged data presented. Statistical difference between treatments were assessed by Holm-Bonferroni adjusted p-values from paired Mann-Whitney test.<br>Nascent RNA-seq experiments were conducted in triplicate, with averaged data presented. Statistical difference between treatments were assessed using EdgeR. Where possible these differences were confirmed by qRT-PCR of total cellular RNA, however the short treatment times used mean that differences visible by nascent RNA-seq are not represented in mature mRNA levels.<br>All attempts at replication were successful.
Randomization	Randomization was not used in this study due to the use of cell lines
Blinding	Investigators were not blinded as this was not relevant to analysis of the data generated here, and the same pipelines and scripts were used to analyze all samples.

Reporting for specific materials, systems and methods

We require information from authors about some types of materials, experimental systems and methods used in many studies. Here, indicate whether each material, system or method listed is relevant to your study. If you are not sure if a list item applies to your research, read the appropriate section before selecting a response.

Materials & experimental systems

n/a	Involved in the study	
☐	☒	Antibodies
☐	☒	Eukaryotic cell lines
☒	☐	Palaeontology
☒	☐	Animals and other organisms
☒	☐	Human research participants
☒	☐	Clinical data

Methods

n/a	Involved in the study	
☐	☒	ChIP-seq
☒	☐	Flow cytometry
☒	☐	MRI-based neuroimaging

Antibodies

Antibodies used

Anti-BRD2 (ChIP-seq): Bethyl A302-582A, lot 1.
Anti-BRD3 (ChIP-seq): Bethyl A302-368A, lot 1.
Anti-BRD4 (ChIP-seq and ChIP-qPCR): Bethyl A301-985A, lot 1.
Anti-BRD4 (Western blotting): Abcam ab128874.
anti-MED1 (ChIP-qPCR): Bethyl A300-793A, lot 2.
anti-MED12 (ChIP-seq and ChIP-qPCR): Bethyl A300-774A, lot 1.
anti-MED26 (ChIP-seq): Bethyl A302-370A, lot 1.
anti-RAD21 (ChIP-seq and ChIP-qPCR): Abcam ab992, lot GR3253930-3.
anti-CTCF (ChIP-qPCR): Millipore 07-729, lot 2922425.
anti-GAPDH (Western blotting): Bethyl A300-641A.

Validation

Anti-BRD2 (ChIP-seq): Bethyl A302-582A, lot 1. Validated for WB and IP in human cell lines on the manufacturer's website. Validated for ChIP in-house by ChIP-qPCR
Anti-BRD3 (ChIP-seq): Bethyl A302-368A, lot 1. Validated for WB and IP in human cell lines on the manufacturer's website. Validated for ChIP in-house by ChIP-qPCR
Anti-BRD4 (ChIP-seq and ChIP-qPCR): Bethyl A301-985A, lot 1. Validated for WB and IP in human cell lines on the manufacturer's website. Validated for ChIP-seq in PMID 28076791
Anti-BRD4 (Western blotting): Abcam ab128874. Validated for WB in human cell lines on the manufacturer's website.
anti-MED1 (ChIP-qPCR): Bethyl A300-793A, lot 2. Validated for WB and IP in human cell lines on the manufacturer's website. Validated for ChIP-seq in PMID 28076791
anti-MED12 (ChIP-seq and ChIP-qPCR): Bethyl A300-774A, lot 1. Validated for WB and IP in human cell lines on the manufacturer's website. Validated for ChIP in-house by ChIP-qPCR
anti-MED26 (ChIP-seq): Bethyl A302-370A, lot 1. Validated for IP in human cell lines on the manufacturer's website. Validated for ChIP in-house by ChIP-qPCR
anti-RAD21 (ChIP-seq and ChIP-qPCR): Abcam ab992, lot GR3253930-3. Validated for ChIP in human cell lines on the manufacturer's website.
anti-CTCF (ChIP-qPCR): Millipore 07-729, lot 2922425. Validated for ChIP in human cell lines on the manufacturer's website.
anti-GAPDH (Western blotting): Bethyl A300-641A. Validated for WB in human cell lines on the manufacturer's website.

Eukaryotic cell lines

Policy information about cell lines

Cell line source(s)	SEM cells were purchased from DSMZ (www.cell-lines.de)
Authentication	Cells were validated by DSMZ by STR DNA typing
Mycoplasma contamination	All cell lines were confirmed to be mycoplasma free
Commonly misidentified lines (See ICLAC register)	None

ChIP-seq

Data deposition

☒ Confirm that both raw and final processed data have been deposited in a public database such as GEO.

☒ Confirm that you have deposited or provided access to graph files (e.g. BED files) for the called peaks.

Data access links
May remain private before publication.

To review GEO accession GSE139437:
Go to https://www.ncbi.nlm.nih.gov/geo/query/acc.cgi?acc=GSE139437

Files in database submission

SEM_BRD2_R1.fastq.gz, SEM_BRD2_R2.fastq.gz
SEM_BRD3_R1.fastq.gz, SEM_BRD3_R2.fastq.gz
SEM_Input_BRD_R1.fastq.gz, SEM_Input_BRD_R2.fastq.gz
SEM_MED12_R1.fastq.gz, SEM_MED12_R2.fastq.gz
SEM_Input_MED12_R1.fastq.gz, SEM_Input_MED12_R2.fastq.gz
SEM_MED26_R1.fastq.gz, SEM_MED26_R2.fastq.gz
SEM_Input_MED26_R1.fastq.gz, SEM_Input_MED26_R2.fastq.gz
SEM_RAD21_R1.fastq.gz, SEM_RAD21_R2.fastq.gz
SEM_Input_RAD21_R1.fastq.gz, SEM_Input_RAD21_R2.fastq.gz
SEM_BRD4_DMSO_R1.fastq.gz, SEM_BRD4_DMSO_R2.fastq.gz
SEM_Input_BRD4_DMSO_R1.fastq.gz, SEM_Input_BRD4_DMSO_R2.fastq.gz
SEM_BRD4_IBET_R1.fastq.gz, SEM_BRD4_IBET_R2.fastq.gz
SEM_Input_BRD4_IBET_R1.fastq.gz, SEM_Input_BRD4_IBET_R2.fastq.gz
SEM_BRD4_DMSO24h_R1.fastq.gz, SEM_BRD4_DMSO24h_R2.fastq.gz
SEM_MED1_DMSO24h_R1.fastq.gz, SEM_MED1_DMSO24h_R2.fastq.gz
SEM_Input_BRD4_MED1_DMSO24h_R1.fastq.gz, SEM_Input_BRD4_MED1_DMSO24h_R2.fastq.gz
SEM_BRD4_AT1_R1.fastq.gz, SEM_BRD4_AT1_R2.fastq.gz
SEM_MED1_AT1_R1.fastq.gz, SEM_MED1_AT1_R2.fastq.gz
SEM_Input_BRD4_MED1_AT1_R1.fastq.gz, SEM_Input_BRD4_MED1_AT1_R2.fastq.gz
SEM_BRD4_UNT_R1.fastq.gz, SEM_BRD4_UNT_R2.fastq.gz
SEM_MED1_UNT_R1.fastq.gz, SEM_MED1_UNT_R2.fastq.gz
SEM_Input_BRD4_MED1_UNT_R1.fastq.gz, SEM_Input_BRD4_MED1_UNT_R2.fastq.gz
SEM_BRD4_HEX_R1.fastq.gz, SEM_BRD4_HEX_R2.fastq.gz
SEM_MED1_HEX_R1.fastq.gz, SEM_MED1_HEX_R2.fastq.gz
SEM_Input_BRD4_MED1_HEX_R1.fastq.gz, SEM_Input_BRD4_MED1_HEX_R2.fastq.gz
SEM_BRD2_peaks.txt.gz, SEM_BRD3_peaks.txt.gz
SEM_MED12_peaks.txt.gz
SEM_MED26_peaks.txt.gz
SEM_RAD21_peaks.txt.gz
SEM_BRD4_DMSO_peaks.txt.gz
SEM_BRD4_IBET_peaks.txt.gz
SEM_BRD4_peaks_AT1_annotated.txt.gz
SEM_MED1_peaks_AT1_annotated.txt.gz
SEM_BRD4_peaks_Hexanediol_annotated.txt.gz
SEM_MED1_peaks_Hexanediol_annotated.txt.gz
SEM_BRD4_peaks_IBET_annotated.txt.gz
SEM_RNA_DMSO90_1_R1.fastq.gz, SEM_RNA_DMSO90_1_R2.fastq.gz, SEM_RNA_DMSO90_2_R1.fastq.gz,
SEM_RNA_DMSO90_2_R2.fastq.gz, SEM_RNA_DMSO90_3_R1.fastq.gz, SEM_RNA_DMSO90_3_R2.fastq.gz
SEM_RNA_IBET90_1_R1.fastq.gz, SEM_RNA_IBET90_1_R2.fastq.gz, SEM_RNA_IBET90_2_R1.fastq.gz,
SEM_RNA_IBET90_2_R2.fastq.gz, SEM_RNA_IBET90_3_R1.fastq.gz, SEM_RNA_IBET90_3_R2.fastq.gz
SEM_RNA_DMSO24h_1_R1.fastq.gz, SEM_RNA_DMSO24h_1_R2.fastq.gz, SEM_RNA_DMSO24h_2_R1.fastq.gz,
SEM_RNA_DMSO24h_2_R2.fastq.gz, SEM_RNA_DMSO24h_3_R1.fastq.gz, SEM_RNA_DMSO24h_3_R2.fastq.gz
SEM_RNA_IBET24h_1_R1.fastq.gz, SEM_RNA_IBET24h_1_R2.fastq.gz, SEM_RNA_IBET24h_2_R1.fastq.gz,
SEM_RNA_IBET24h_2_R2.fastq.gz, SEM_RNA_IBET24h_3_R1.fastq.gz, SEM_RNA_IBET24h_3_R2.fastq.gz
SEM_RNA_Untreated_1_R1.fastq.gz, SEM_RNA_Untreated_1_R2.fastq.gz, SEM_RNA_Untreated_2_R1.fastq.gz,
SEM_RNA_Untreated_2_R2.fastq.gz, SEM_RNA_Untreated_3_R1.fastq.gz, SEM_RNA_Untreated_3_R2.fastq.gz
SEM_RNA_Hexanediol_1_R1.fastq.gz, SEM_RNA_Hexanediol_1_R2.fastq.gz, SEM_RNA_Hexanediol_2_R1.fastq.gz,
SEM_RNA_Hexanediol_2_R2.fastq.gz, SEM_RNA_Hexanediol_3_R1.fastq.gz, SEM_RNA_Hexanediol_3_R2.fastq.gz
SEM_RNA_IBET90_hg19_featuresCounted.txt.gz, SEM_RNA_IBET90_hg19_contrast_cpm.txt.gz
SEM_RNA_IBET24h_hg19_featuresCounted.txt.gz, SEM_RNA_IBET24h_hg19_contrast_cpm.txt.gz
SEM_RNA_Hexanediol_hg19_featuresCounted.txt.gz, SEM_RNA_Hexanediol_hg19_contrast_cpm.txt.gz
SEM_CapC_DMSO90_1_R1.fastq.gz, SEM_CapC_DMSO90_1_R2.fastq.gz, SEM_CapC_DMSO90_2_R1.fastq.gz,
SEM_CapC_DMSO90_2_R2.fastq.gz, SEM_CapC_DMSO90_3_R1.fastq.gz, SEM_CapC_DMSO90_3_R2.fastq.gz
SEM_CapC_IBET90_1_R1.fastq.gz, SEM_CapC_IBET90_1_R2.fastq.gz, SEM_CapC_IBET90_2_R1.fastq.gz,
SEM_CapC_IBET90_2_R2.fastq.gz, SEM_CapC_IBET90_3_R1.fastq.gz, SEM_CapC_IBET90_3_R2.fastq.gz
SEM_CapC_DMSO24h_1_R1.fastq.gz, SEM_CapC_DMSO24h_1_R2.fastq.gz, SEM_CapC_DMSO24h_2_R1.fastq.gz,
SEM_CapC_DMSO24h_2_R2.fastq.gz, SEM_CapC_DMSO24h_3_R1.fastq.gz, SEM_CapC_DMSO24h_3_R2.fastq.gz
SEM_CapC_IBET24h_1_R1.fastq.gz, SEM_CapC_IBET24h_1_R2.fastq.gz, SEM_CapC_IBET24h_2_R1.fastq.gz,
SEM_CapC_IBET24h_2_R2.fastq.gz, SEM_CapC_IBET24h_3_R1.fastq.gz, SEM_CapC_IBET24h_3_R2.fastq.gz
SEM_CapC_DMSO90-JQ1_1_R1.fastq.gz, SEM_CapC_DMSO90-JQ1_1_R2.fastq.gz, SEM_CapC_DMSO90-JQ1_2_R1.fastq.gz,
SEM_CapC_DMSO90-JQ1_2_R2.fastq.gz, SEM_CapC_DMSO90-JQ1_3_R1.fastq.gz, SEM_CapC_DMSO90-JQ1_3_R2.fastq.gz
SEM_CapC_JQ1_90_1_R1.fastq.gz, SEM_CapC_JQ1_90_1_R2.fastq.gz, SEM_CapC_JQ1_90_2_R1.fastq.gz,
SEM_CapC_JQ1_90_2_R2.fastq.gz, SEM_CapC_JQ1_90_3_R1.fastq.gz, SEM_CapC_JQ1_90_3_R2.fastq.gz
SEM_CapC_Untreated_1_R1.fastq.gz, SEM_CapC_Untreated_1_R2.fastq.gz, SEM_CapC_Untreated_2_R1.fastq.gz,
SEM_CapC_Untreated_2_R2.fastq.gz, SEM_CapC_Untreated_3_R1.fastq.gz, SEM_CapC_Untreated_3_R2.fastq.gz
SEM_CapC_Hexanediol_1_R1.fastq.gz, SEM_CapC_Hexanediol_1_R2.fastq.gz, SEM_CapC_Hexanediol_2_R1.fastq.gz,
SEM_CapC_Hexanediol_2_R2.fastq.gz, SEM_CapC_Hexanediol_3_R1.fastq.gz, SEM_CapC_Hexanediol_3_R2.fastq.gz
SEM_CapC_DMSO24h-AT1_1_R1.fastq.gz, SEM_CapC_DMSO24h-AT1_1_R2.fastq.gz, SEM_CapC_DMSO24h-AT1_2_R1.fastq.gz, SEM_CapC_DMSO24h-AT1_2_R2.fastq.gz, SEM_CapC_DMSO24h-AT1_3_R1.fastq.gz,

	SEM_CapC_DMSO24h-AT1_3_R2.fastq.gz<br>SEM_CapC_AT1_1_R1.fastq.gz, SEM_CapC_AT1_1_R2.fastq.gz, SEM_CapC_AT1_2_R1.fastq.gz, SEM_CapC_AT1_2_R2.fastq.gz, SEM_CapC_AT1_3_R1.fastq.gz, SEM_CapC_AT1_3_R2.fastq.gz<br>SEM_IBET90_CapC_combined.gfc.gz<br>SEM_IBET24h_CapC_combined.gfc.gz<br>SEM_JQ1_CapC_combined.gfc.gz<br>SEM_Hexanediol_CapC_combined.gfc.gz<br>SEM_AT1_CapC_combined.gfc.gz
Genome browser session (e.g. UCSC)	https://genome.ucsc.edu/s/ncrump/Crump_BET1<br>https://genome.ucsc.edu/s/ncrump/Crump_AT1

Methodology

Replicates	One replicate for ChIP-seq datasets. Three biological replicates for all Capture-C and nascent RNA-seq conditions.
Sequencing depth	BRD2: total reads: 58899616, uniquely mapped reads: 44273608, read length: 40 bp, Paired end<br>BRD3: total reads: 54105972, uniquely mapped reads: 43954644, read length: 40 bp, Paired end<br>BRD2/3 Input: total reads: 117927878, uniquely mapped reads: 97923080, read length: 40 bp, Paired end<br>BRD4 DMSO: total reads: 85061280, uniquely mapped reads: 56910726, read length: 40 bp, Paired end<br>BRD4 DMSO Input: total reads: 28348322, uniquely mapped reads: 15444772, read length: 40 bp, Paired end<br>BRD4 IBET total reads: 81260702, uniquely mapped reads: 54061628, read length: 40 bp, Paired end<br>BRD4 IBET Input total reads: 65060178, uniquely mapped reads: 47650290, read length: 40 bp, Paired end<br>MED12: total reads: 9184289, uniquely mapped reads: 8498648, read length: 40 bp, Paired end<br>MED12 Input: total reads: 8846506, uniquely mapped reads: 8546638, read length: 40 bp, Paired end<br>MED26: total reads: 1479242, uniquely mapped reads: 1430252, read length: 40 bp, Paired end<br>MED26 Input: total reads: 60070334, uniquely mapped reads: 34517722, read length: 40 bp, Paired end<br>RAD21: total reads: 49958214, uniquely mapped reads: 38869774, read length: 40 bp, Paired end<br>RAD21 Input: total reads: 12498964, uniquely mapped reads: 9843406, read length: 40 bp, Paired end<br>BRD4 DMSO24h: total reads: 65202758 uniquely mapped reads: 44854656 read length: 40 bp, Paired end<br>MED1 DMSO24h: total reads: 73123840 uniquely mapped reads: 50348158 read length: 40 bp, Paired end<br>BRD4/MED1 Input DMSO24h: total reads: 8876208 uniquely mapped reads: 2977834 read length: 40 bp, Paired end<br>BRD4 AT1 24h: total reads: 57806620 uniquely mapped reads: 39108868 read length: 40 bp, Paired end<br>MED1 AT1 24h: total reads: 70255900 uniquely mapped reads: 47805198 read length: 40 bp, Paired end<br>BRD4/MED1 Input AT1 24h: total reads: 10922086 uniquely mapped reads: 3633274 read length: 40 bp, Paired end<br>BRD4 untreated: total reads: 57161236 uniquely mapped reads: 38165984 read length: 40 bp, Paired end<br>MED1 untreated: total reads: 67805594 uniquely mapped reads: 46136904 read length: 40 bp, Paired end<br>BRD4/MED1 Input untreated: total reads: 15863394 uniquely mapped reads: 8246944 read length: 40 bp, Paired end<br>BRD4 hexanediol 30': total reads: 102111230 uniquely mapped reads: 67983530 read length: 40 bp, Paired end<br>MED1 hexanediol 30': total reads: 68687368 uniquely mapped reads: 45613110 read length: 40 bp, Paired end<br>BRD4/MED1 Input hexanediol 30': total reads: 11437302 uniquely mapped reads: 4701398 read length: 40 bp, Paired end
Antibodies	Anti-BRD2: Bethyl A302-582A, lot 1<br>Anti-BRD3: Bethyl A302-368A, lot 1<br>Anti-BRD4: Bethyl, A301-985A, lot 1<br>anti-MED1: Bethyl, A300-793A, lot 9<br>anti-MED12: Bethyl A300-779A, lot 1<br>anti-MED26: Bethyl A302-370A, lot 1<br>anti-RAD21: Abcam ab992, lot GR3253930-3
Peak calling parameters	Peaks were called using the Homer tool findPeaks, with the input track provided for background correction, using the -style histone or -style factor options to call peaks in histone modification or transcription factor/ATAC datasets, respectively.
Data quality	Reads were filtered to remove PCR duplicates. Called peaks were analyzed as described on homer.ucsd.edu and compared to input track, with a threshold of FDR < 0.001 applied.
Software	Quality control of FASTQ reads, genome alignment, PCR duplicate filtering, blacklisted region filtering and UCSC data hub generation was performed using an in-house pipeline: https://github.com/Hughes-Genome-Group/NGseqBasic/releases
